# Errata

**DOI:** 10.36660/abc.20220846

**Published:** 2022-11-23

**Edição de Setembro de 2020, vol. 115(3), págs. 478-479**

No minieditorial “Consumo de Oxigênio e Aptidão Cardiorrespiratória | Diferença entre Idade Cronológica e Biológica”, com número de DOI: https://doi.org/10.36660/abc.20200582, publicado no periódico Arquivos Brasileiros de Cardiologia, 115(3): 478-479, na página 478, corrigir o DOI para: https://doi.org/10.36660/abc.20200648

**Edição de Julho de 2022, vol. 119 (1), págs. 143-211**

Na “Diretriz de Miocardites da Sociedade Brasileira de Cardiologia – 2022”, com número de DOI: https://doi.org/10.36660/abc.20220412, publicado no periódico Arquivos Brasileiros de Cardiologia, 119(1):143-21, nas páginas 143 e 147, corrigir o nome do autor Marcelo Imbroise Bittencourt para: Marcelo Imbroinise Bittencourt.

**Edição de Outubro de 2022, vol. 119 (4), págs. 638-680**

Na “Diretriz da Sociedade Brasileira de Cardiologia sobre a Análise e Emissão de Laudos Eletrocardiográficos – 2022”, com número de DOI: https://doi.org/10.36660/abc.20220623, publicado no periódico Arquivos Brasileiros de Cardiologia, 119(4):638-680, na página 655 (Item 6.1.4.2 Índice de Sokolow Lyon), corrigir a frase “Não deve ser utilizado em atletas” para: “Isoladamente, não deve ser utilizado em atletas.”

Na página 666 (Item 12.1.2 Achados Eletrocardiográficos Atuais (Grupo 2)), corrigir a frase “Duração do QRS ≥ 160 ms” para: “Duração do QRS ≥ 140ms;”.

**Edição de Dezembro de 2022, vol. 119(6), págs. 883-890**

No artigo original “Tortuosidade das Artérias Coronárias como um Novo Fenótipo para Isquemia sem Doença Arterial Coronariana”, com número de DOI: https://doi.org/10.36660/abc.20210787, publicado no periódico Arquivos Brasileiros de Cardiologia, 119(6): 883-890, na página 884, a figura correta encontra-se no link: http://abccardiol.org/supplementary-material/2022/11906/2021_0787_fig-01 corrigida.jpg

