# Supplementary material for: Errata
Source: Arq Bras Cardiol. 2022 Nov 23;119(6):1008. [Article in Portuguese] doi: 10.36660/abc.20220846 (PMC9814798; doi:10.36660/abc.20220846)
Supplement: Translation

# Translation: Erratum

**September 2020 Issue, vol. 115(3), pages 478-479**

In the Short Editorial “Oxygen Consumption and Cardiorespiratory Fitness. The Difference between Chronological and Biological Age”, with DOI: https://doi.org/10.36660/abc.20200582, published in the journal Arquivos Brasileiros de Cardiologia, 115(3): 478-479, in page 478, correct the DOI for: https://doi.org/10.36660/abc.20200648

**July 2022 Issue, vol. 119 (1), pages 143-211**

In the “Brazilian Society of Cardiology Guideline on Myocarditis – 2022”, with DOI number: https://doi.org/10.36660/abc.20220412, published in the journal Arquivos Brasileiros de Cardiologia, 119(1):143-21, on pages 143 and 147, correct the name of the author Marcelo Imbroise Bittencourt to: Marcelo Imbroinise Bittencourt.

**October 2022 Issue, vol. 119 (4), pages 638-680**

In the “ Brazilian Society of Cardiology Guidelines on the Analysis and Issuance of Electrocardiographic Reports – 2022”, with DOI number: https://doi.org/10.36660/abc.20220623, published in the journal Arquivos Brasileiros de Cardiologia, 119(4):638-680, on page 655 (Item 6.1.4.2 Sokolow-Lyon Index), correct the phrase “This index should not be used in athletes” to: “Alone, this index should not be used in athletes.”

On page 665 (Item 12.1.2 Abnormal ECG Findings (Group 2)), correct the phrase “QRS duration ≥ 160 ms” to: “QRS duration ≥ 140 ms;”.

**December 2022 Issue, vol. 119(6), pages 883-890**

In the Original Article “Coronary Tortuosity as a New Phenotype for Ischemia without Coronary Artery Disease”, with DOI: https://doi.org/10.36660/abc.20210787, published in the journal Arquivos Brasileiros de Cardiologia, 119(6): 883-890, in page 884, the correct figure is in the link: http://abccardiol.org/supplementary-material/2022/11906/2021_0787_fig-01corrigida.jpg
